# Faeces – Urine separation via settling and displacement: Prototype tests for a novel non-sewered sanitation system

**DOI:** 10.1016/j.scitotenv.2020.141881

**Published:** 2021-01-20

**Authors:** Jan Hennigs, Kristin T. Ravndal, Alison Parker, Matt Collins, Ying Jiang, Athanasios J. Kolios, Ewan McAdam, Leon Williams, Sean Tyrrel

**Affiliations:** a School of Water, Energy and Environment, Cranfield University, United Kingdom; b Freeform Design & Innovation Ltd., Flitwick, United Kingdom; c University of Strathclyde, Glasgow, United Kingdom

**Keywords:** ANOVA, analysis of variance, COD, chemical oxygen demand, DOE, design of experiments, F, frequency (of defecation), NMT, nano membrane toilet, sCOD, soluble chemical oxygen demand, TP, toilet paper, TS, total solids, UDDT, urine diversion dehydration toilet, V, (tank) volume, VS, volatile solids, Prototyping, Testing, DOE

## Abstract

The development of novel, non-sewered sanitation systems like the Nano Membrane Toilet requires thorough investigation of processes that may seem well-understood. For example, unlike the settling of primary sludge, the separation of solids from liquids in a small-volume container at the scale of a household toilet has not been studied before. In two sets of experiments, the settling of real faeces and toilet paper in settling columns and the settling of synthetic faeces in a conical tank are investigated to understand the factors affecting the liquid quality for downstream treatment processes. Toilet paper is found to be a major inhibitor to settling of solids. While a lower overflow point results in better phase separation through displacement of liquid, a higher overflow point and frequent removal of solids may be more advantageous for the liquid quality.

## Highlights

• Faeces and toilet paper do not settle in urine within small volume tank.
• Toilet paper keeps faeces in suspension.
• Phase separation can be achieved through displacement of liquid.
• Liquid quality decreases over time and with higher solids-liquids ratio in tank.

## Graphical abstract

**Figure:**
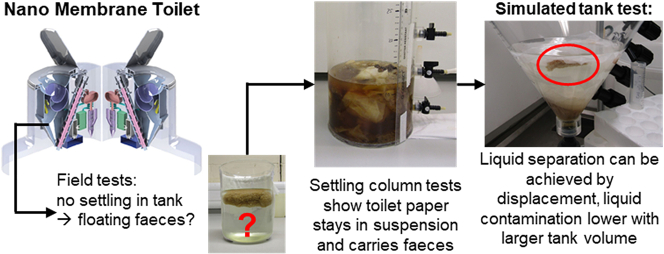

## 1 Introduction

**Fig. 1:**
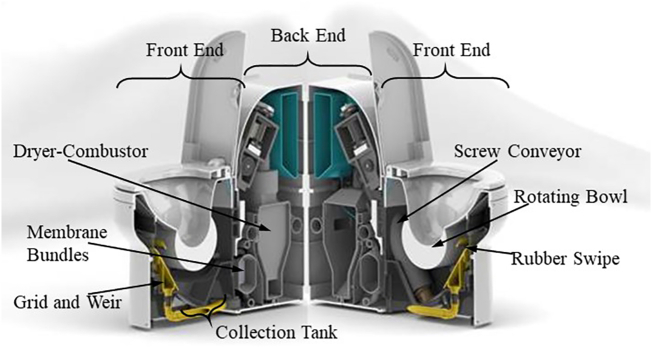
Schematic of the nano membrane toilet.

In an effort to develop a safe, waterless, non-sewered sanitation technology, and in response to the Bill & Melinda Gates Foundation's *Reinvent The Toilet Challenge* (Bill and Melinda Gates Foundation, 2013), Cranfield University is developing the Nano Membrane Toilet (NMT) (Parker, 2014), a novel product intended for household sizes of up to 10 people (Fig. 1). It features a waterless mechanical flush with a rotating bowl that receives faeces, urine, and toilet paper and transports it into a conical collection tank with about 10 l volume underneath the bowl (J. Hennigs et al., 2019). From this tank, liquid waste flows through a grid and over a weir, from where it enters a membrane treatment process (Kamranvand et al., 2018). Solid waste is transported out of the tank via screw conveyance, followed by drying and combustion. Heat from the combustion is recovered to power the membrane process (Onabanjo et al., 2016a). The Flush, membrane and combustion processes have been developed and tested to work individually, while transport and dewatering of solids via auger are still under investigation.

Existing solutions of separating liquids from solids at the source, so-called urine diversion dehydration toilets (UDDT), have been shown to be problematic for users (Mkhize et al., 2017; Roma et al., 2013), and field trials of the NMT's mechanical flush revealed that users of UDDT preferred the flush over their usual toilets (Jan Hennigs et al., 2019). Similarly, other studies reported problems with using, operating, and maintaining urine diversion toilets (Blume and Winker, 2011; Lienert and Larsen, 2010; Uddin et al., 2014). Therefore, a combined collection of liquids and solids is the desired mode of operation in the NMT. This requires post-flush phase separation before both waste streams can be introduced to their respective treatment processes. With better separation of liquid from solid waste streams, the performance of the membrane treatment processes would likely increase (Kamranvand et al., 2018; Sun and Leiknes, 2012), as would the amount of heat recovered from the combustion (Onabanjo et al., 2016b). Research into the transport of solids via auger includes dewatering in the auger, and it might become apparent that a certain amount of liquid is required for the auger to perform optimally (Mercer et al., 2016). However, we expect that solid-liquid separation will have to occur mainly in the NMT's collection tank. In the original concept design of the NMT, this separation was intended to be achieved by settling of solids: Over the course of one or more days, the tank would fill with faeces, toilet paper and urine, and the solid faeces and toilet paper were thought to settle to the bottom of the tank. Preliminary investigations of settling behaviour of faeces in water showed that even those faeces that initially floated eventually sank (Cruddas et al., 2015). This was explained by the fact that faeces that float do so because of their gas content (Levitt and Duane, 1972), and once the gas exited the faeces, they would settle. However, during field tests in South Africa, in which real faeces, urine, and toilet paper where collected in a prototype of the collection tank, settling did not seem to occur (unpublished results). This was a significant observation, as it challenged a fundamental expectation of how the NMT would operate. Since the prototype used in these field tests was not transparent, the actual settling behaviour of faeces and toilet paper in urine could not be observed. Unfortunately, there is no literature describing sufficiently similar settling processes.

Gravity settling and thickening are comparably well-understood processes in treatment of wastewater (Metcalf and Eddy Inc. et al., 2014) and, to a lesser degree, of faecal sludge (Dodane and Bassan, 2014), but the influent of settling tanks of municipal scale is vastly different from the fresh urine, faeces, and toilet paper in a small volume container. At the time at which wastewater or faecal sludge reach treatment facilities, toilet paper and faeces have disintegrated into much smaller particles due to the mechanical effects of transport through a sewer network and/or pumping, creating a more homogenous slurry or sludge (Eren and Karadagli, 2012; Niwagaba et al., 2014) than the contents of the NMT's collection tank. Thus, calculations for the design of settling tanks are based on small, ideally spherical particles (Bassan et al., 2014), and on solids content measurements in *Imhoff cones*, for which coarse materials are usually removed (Metcalf and Eddy Inc. et al., 2014). In contrast, toilet paper and faeces in the NMT collection tank appeared to be mainly still intact. Even the settling processes in septic tanks – potentially the closest analogue to the NMT's collection tank – are rarely the object of academic research. In one recent example, Dos Santos et al. (2017) describe an upflow anaerobic sludge blanket reactor as an improvement of the classic septic tank to enhance settling. It had greater organic matter removal efficiency than a traditional septic tank. Generally, guidance about septic tanks just assumes settling of solids, but does not appear to be investigated thoroughly (Oxfam, 2008; Tilley et al., 2014). Investigations on the settling behaviour of faeces and intact toilet paper in a small volume container could therefore not only inform the development of the Nano Membrane Toilet, but also provide yet-unreported information for the better understanding of settling processes in faecal sludge treatment facilities, septic tanks and other small volume sanitation systems, for example to be found in container-based sanitation (Tilmans et al., 2015)

**Fig. 2:**
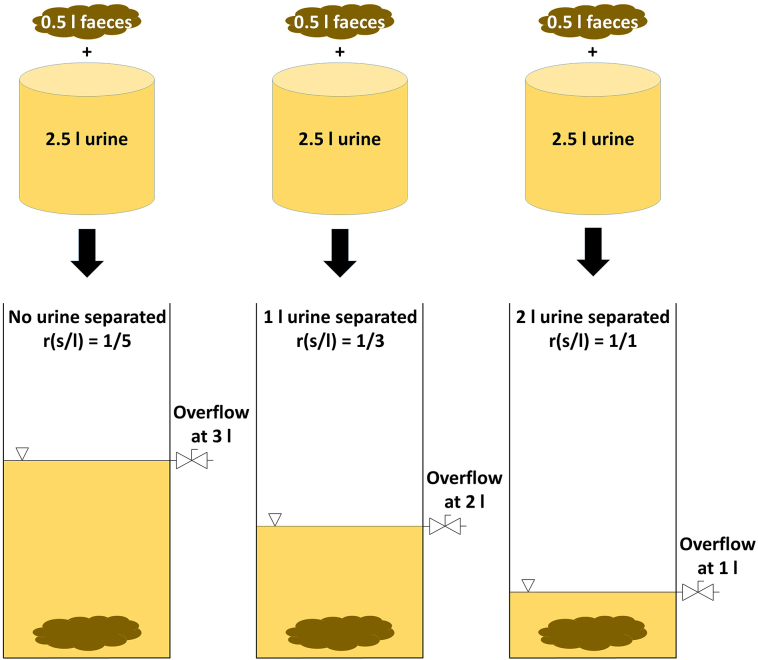
Solid-liquid separation through displacement: the lower the liquid overflow point of the collection tank, the more urine is separated through displacement by exiting through the overflow. This promotes a higher solids-to-liquids ratio.

Additionally, the observations in the field prompted the idea to achieve phase separation by settling and displacement, rather than settling alone: With a lower overflow point, a higher ratio of solids to liquids in the tank would be promoted. As new solids are introduced, liquids are displaced out of the tank (Fig. 2). Thus, a better separation of liquids from the tank and consequently a better phase separation is achieved. To test the practicability of phase separation by displacement for the NMT and similar small-volume sanitation systems, the effectiveness would have to be investigated in a tank with a similar (conical) geometry to that of the NMT collection tank. The conical shape of the tank was chosen as the sloped walls promote settling-thickening, which is the reason municipal thickeners and *Imhoff tanks* often take this shape (Dodane and Bassan, 2014; Metcalf and Eddy Inc. et al., 2014). The success of phase separation is determined not only by the ratio of solids to liquids in the collection tank, but also by the quality of the overflowing liquid: a higher pollution of small solids particles and soluble organic compounds, represented by the Chemical Oxygen Demand (COD), in the overflowing liquid would negatively affect the downstream membrane treatment processes (Kamranvand et al., 2018).

Based on observations from the settling column tests, three factors were suspected to most likely have an effect on the quality of overflowing liquid: First, the size of the collection tank's maximum liquid volume (V, determined by the tank's geometry and the height of the overflow) determines the ratio of solids to liquids in the tank (Fig. 2), and consequently the amount of liquid in contact with solids. Under the assumption that the transfer of particles and organic compounds into the liquid is relatively independent from the solids-liquids ratio, a higher concentration of both would be expected in the liquid if less liquid is available. Thus, it would be expected that a smaller tank volume will yield a higher concentration of solids and COD in the liquid phase. Second, the frequency with which solids are added (F) was expected to affect how much time the processes within the tank are given to return from dynamic to relatively static. The more often faeces are added to the system, the more often disturbance will occur and that likely promotes transfer of small particles and COD into the liquid. The third factor under consideration was whether or not toilet paper was added along with the faeces (TP). As was already observed in previous field trials, and again in the settling column tests (see results section below), toilet paper appears to keep faeces from settling, regardless of faeces-density. The more faeces are kept in suspension, the higher the solid-liquid interface, along which particles and COD could be transferred.

This study investigates the settling of fresh faeces and toilet paper in urine, and whether a lower liquid overflow level promotes solid-liquid separation within small volume settling columns. Furthermore, the study tests the hypothesis that a smaller tank volume, a higher frequency of adding faeces, and the addition of toilet paper, individually or in combination, contribute to a higher concentration of small solids particles and COD in the overflowing liquid in a conical prototype collection tank for the Nano Membrane Toilet.

## 2 Materials and methodology

The effectiveness of solid-liquid separation can not only be gauged by the visual observation of less liquid in the tank, but also in the quality of the overflowing liquid. To gauge the concentration of particles and organic compounds, Total Solids (TS) and COD were measured, respectively (Metcalf and Eddy Inc. et al., 2014).

### 2.1 Tests in settling column

**Fig. 3:**
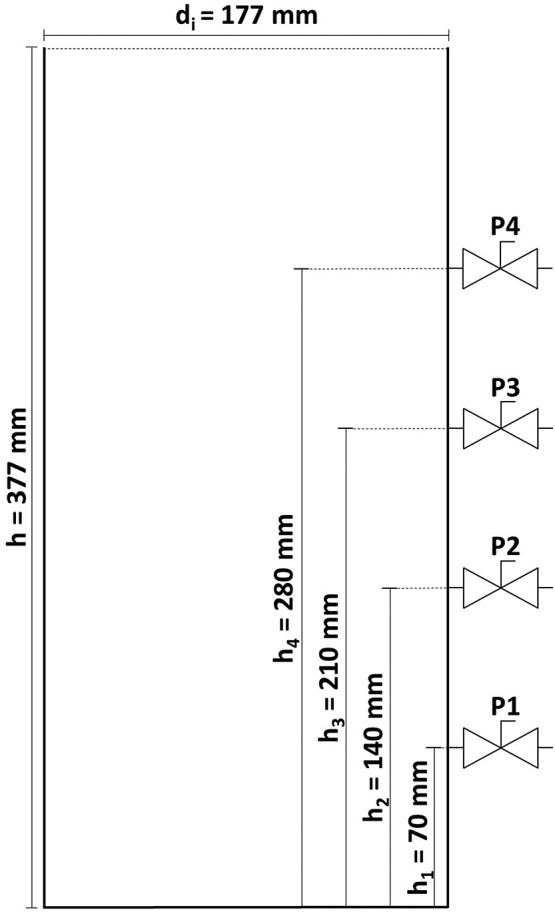
Schematic of transparent settling columns used in real-faeces tests.

Following the observations during previous field tests, the settling behaviour of faeces was investigated in settling tests in transparent columns (Fig. 3), using real faeces and urine, with the addition of toilet paper. Urine and faeces samples were anonymous donations collected from users of the office toilets at Cranfield Water Science Institute.

With an inner diameter of 177 mm and a height of 377 mm, the columns have a maximum volume of 9.28 l. The columns have four sampling ports to draw liquid samples at 70, 140, 210, and 280 mm height respectively. The ports were numbered P1 – P4 from lowest to highest.

Simulating the use of the NMT by several people, three columns were filled with fresh urine, faeces, and toilet paper consecutively, over the course of five days for the first two columns and four days for the third. The numbers of simulated urinations and defecations, and their timing, were subject to the time and numbers of donations of fresh faeces and urine, but it was attempted to maintain a ratio of 3 urination events per 1 defecation event, with peak times in the early morning and early afternoon hours. On average, a single defecation weighed 120.3 g, and a single urination averaged 125.8 g. The considerations of time and weight of toilet uses were based on data gathered by Cranfield researchers (Cruddas et al., 2015). The numbers of defecation and urination events during a given hour for each day of the three column tests can be found in Table a in the supplementary material. Figure a in the supplementary material shows the average daily toilet use events simulated over the course of a day for all columns. To investigate whether displacement of urine could improve solid-liquid separation, the liquid-overflow points for the three columns were set at different heights: For the first column test (C1), the highest sampling port (P4) remained open and thus acted as an overflow. For the second column test (C2), the third sampling port from the bottom (P3) remained open, and for the third column test (C3) the second port from the bottom (P2).

During the workday, at least hourly photographs were taken of the column. Liquid samples (about 30 ml each) were taken from all sampling ports below filling level three times per day. As indicators of liquid quality, COD, soluble COD (sCOD, obtained by filtering the sample with a 450 nm pore size syringe filter before analysis), TS, and volatile solids (VS) were determined for each sample, using standard methods.

### 2.2 Tests in prototype conical collection tank

To better understand the processes in a collection tank as it would be found in the NMT, controlled experiments in a prototype conical tank were conducted. Since the main objective of these experiments was the investigation of the influence of three factors on the overflow liquid quality, these factors had to be the only variables affecting the liquid quality within the tank. Therefore, using real faeces and urine was unfeasible, as both vary too much in their physical and chemical makeup (Rose et al., 2015). Consequently, synthetic faeces and water were used for this set of experiments. Following a Design of Experiments (DOE) approach (Montgomery, 2009), a 2^3^ full factorial design was chosen to investigate the three factors.

#### 2.2.1 2^3^ – factorial experimental design

The three factors to be investigated were the suspected factors to influence liquid quality: The tank's maximum liquid volume (V), the loading Frequency (F), and the addition of toilet paper (TP).

Toilet paper absorbs liquid and adds to the solids-content in the tank. The tank volume limits in which space toilet paper can suspend faeces. The frequency at which faeces are added may affect settling and suspension of faeces through toilet paper. Therefore, it is conceivable that the liquid quality would also be affected by interactions of the three factors under consideration. In order to investigate all three factors as well as their interactions, a full factorial experimental design with two levels per factor was developed (Montgomery, 2009). Table 1 shows the eight possible combinations of levels for the three factors. The runs were carried out in triplicate, in random order. The high and low levels for the variables were set as:

- tank volume (V): low (−) = 0.7 l; high (+) = 2 l
- loading frequency (F): low (−) = 1 flush event per two hours (1/2 h); high (+) = 1 flush event per hour (1/h)
- toilet paper (TP): low (−) = no toilet paper; high (+) = toilet paper added.

**Table 1:** Full factorial design for liquid displacement tests. There are eight different combinations of three factors at two levels.

Combination/Factor	1	2	3	4	5	6	7	8
Tank volume (V)	+	+	+	+	−	−	−	−
Loading frequency (F)	+	+	−	−	+	+	−	−
Toilet paper (TP)	+	−	+	−	+	−	+	−

#### 2.2.2 Experimental rig

**Fig. 4:**
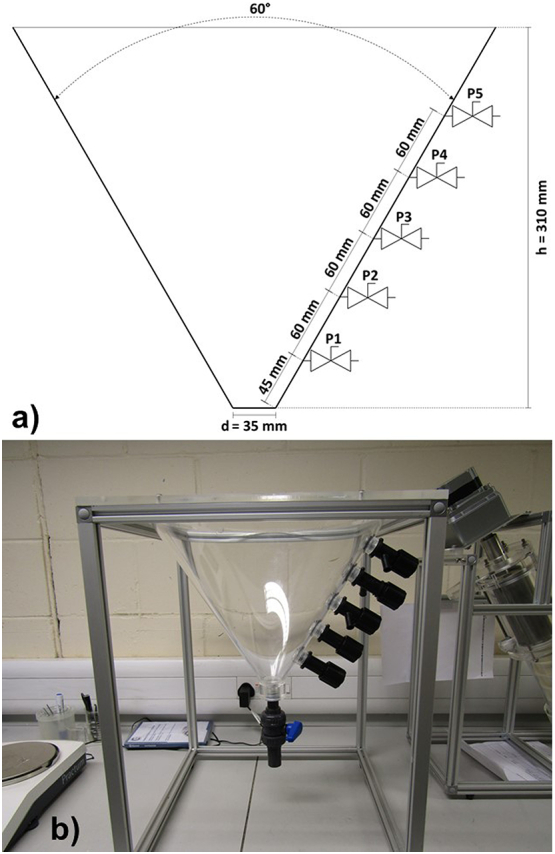
a) Technical drawing of conical collection tank; b) Photo of experimental rig with five sampling ports. Only ports two and three were used for the experiments.

The prototype tank used for this study was designed to resemble the collection tank of the NMT (Fig. 4). It is a conical tank with a 60° angle, which is used in standard inclined settlers (Metcalf and Eddy Inc. et al., 2014), a maximum filling height of 310 mm, and five sampling ports along the side (P1–P5 from lowest to highest). A larger valve at the bottom allowed for quick emptying of the tank. As the sampling ports are equidistant to each other on the inclining wall of a conical tank, the volume underneath the port increases exponentially with each port. If P2 is used as overflow, the maximum filling volume is 0.7 l, including the small volume below the bottom of the tank and the closed valve. If P3 is used, the maximum filling volume is 2 l.

#### 2.2.3 Synthetic faeces

To ensure that the high variability of real faeces (Rose et al., 2015) did not affect the experiment, a synthetic simulant was used instead. Penn et al. (2018) discuss various recipes for synthetic faeces and combine them to develop a simulant with high physical and chemical similarity to real faeces. Their recipe was amended in one aspect: Rather than oleic acid, an ingredient that was originally used by Kaba et al. (1990) who seem to have chosen it arbitrarily as lipid and without explaining their choice, we used peanut oil. With our decision we follow Wignarajah et al. (2006), who also used peanut oil, explaining their choice with its high content of oleic acid. Since the original choice of oleic acid was arbitrary, but both oleic acid and peanut oil have been reported to produce good results (Penn et al., 2018), we chose the cheaper alternative.

To simulate both normal stool and soft stool/diarrhoea, simulants were produced with 65% and 80% water content respectively. Both consistencies were also produced with additional baker's yeast, to produce floating stools (Figure b, supplementary material). A weekly batch of synthetic faeces with 65% water content without baker's yeast was produced. From this, the four samples were created by adding water and baker's yeast as necessary.

#### 2.2.4 Experimental procedure

To be able to investigate the three factors V, F, and TP in isolation, a simplified procedure was followed that ignored the addition of urine and assumed the tank was filled with liquid to weir-height at the beginning of each experiment. This simulated the Nano Membrane Toilet in operation once the tank has initially been filled with urine.

Before each run, the tank was cleaned thoroughly with soap and rinsed with tap water. It was then filled with tap water to the level of the sampling port used as overflow (P2 for the low-level volume, P3 for high-level volume). Over the course of the experimental run, four samples of synthetic faeces were then added to the tank, each weighing 50 g. For each run, there were two samples with 65% and 80% water content, respectively, one with and one without additional baker's yeast. The samples were added in random order, one every hour (F = 1/h), or every 2 h (F = 1/2 h), depending on the level set for F. If the level for TP was “+”, six sheets of toilet paper were added to each faeces sample. The liquid overflowing from the open sampling port (about 30–50 ml) was collected and analysed for TS, COD, and sCOD. Photographs were taken of the transparent tank before and after each addition of synthetic faeces. With four liquid samples, there were four data points available for each parameter for each individual run. For each parameter and run, a linear fit of the four data points was calculated, and the slope of the fit function was used as the dependent variable in a three way analysis of variance (ANOVA) (Montgomery, 2009), using SPSS software. This way, the effects of V, TP, F, and their interactions could be analysed without having to consider the effect of time.

### 2.3 Ethics statement

The methodologies used for this study were approved by the Cranfield University Research Ethics Committee (reference numbers CURES/4982/2018 CURES/2310/2017, CURES/3245/2017, & CURES/3242/2017 for the experiments using real faeces and urine in a settling column; and CURES/7913/2019 for the experiments using simulant faeces in a conical tank prototype). Donors of urine and faeces could donate anonymously and were instructed to do so only after signing a consent form, supplied to them alongside an information sheet inside the toilet cubicle.

## 3 Results

### 3.1 Settling column tests

#### 3.1.1 Visual observations

All three column tests yielded the observation that the faeces – toilet paper mixture did not settle well in urine. Figure c in the supplementary material shows the three columns at the point of solids reaching the overflow level, and large *pockets* of liquid are visible underneath the solids. It does, however, appear that eventually, a layer of faeces-toilet paper mixture covered the entire cross-sectional area of the column, creating a *plug*, preventing any liquid below from exiting. Thus, this trapped liquid could not be displaced, unlike the liquid added after the creation of the plug, which drained through the open sampling port. All sampling ports were quickly covered with solids, resulting in very slow outflow velocity. For sampling from the lower ports, a small piece of wire was poked through the port to encourage quicker outflow of liquid. Even where the sampling ports were covered by the apparent plug, liquid samples could still be collected, hence some liquid still flowed through the apparent solids plug. The open sampling port, acting as overflow point, was left untouched, and the liquid slowly drained into an adjacent container.

#### 3.1.2 Liquid sample measurements

**Fig. 5:**
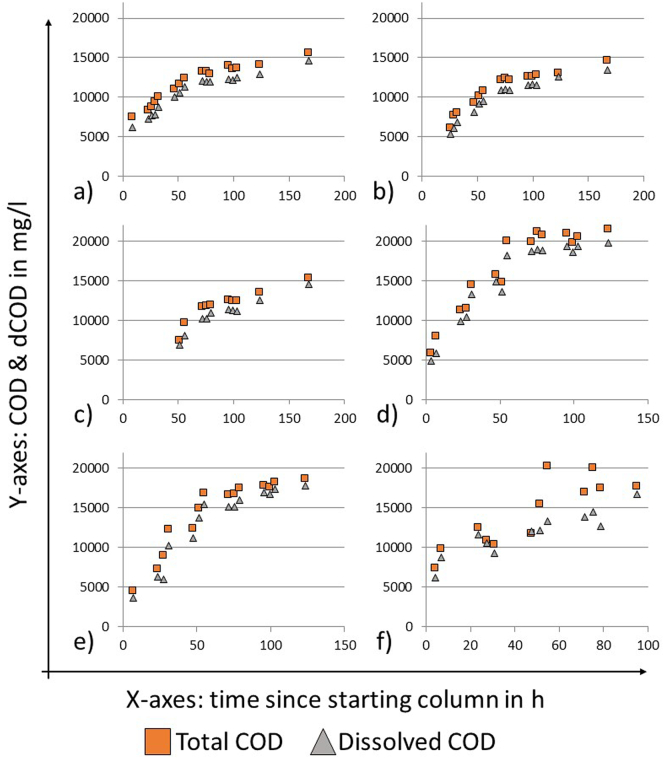
COD and sCOD measurements for settling column tests. Samples were taken from the sampling ports on the side of the column. a) Column 1, sampling port 3; b) Column 1, sampling port 2, c) Column 1, sampling port 1, d) Column 2, sampling port 2, e) Column 2, sampling port 1, f) Column 3, sampling port 1.

Both the COD and sCOD concentrations rose steadily over time for samples taken from all sampling ports (Fig. 5). While the increase is quite steep during the first 48 h after taking the first sample, it appears to plateau slightly after this time. This is true for all sampling ports, meaning that the COD concentrations at a higher point of the column increase more rapidly during the first 48 h even when the concentrations at the lower sampling ports are already plateauing (e.g. compare Fig. 5 a) vs c)). This could be an indication of limited vertical mixing of the liquid.

**Fig. 6:**
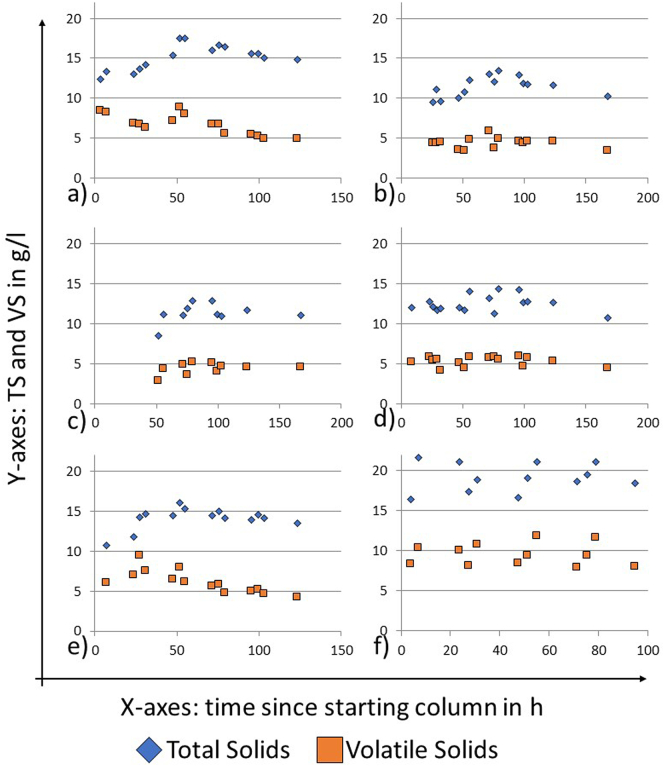
TS and VS measurements for settling column tests. Samples were taken from the sampling ports on the side of the column. a) Column 1, sampling port 3; b) Column 1, sampling port 2, c) Column 1, sampling port 1, d) Column 2, sampling port 2, e) Column 2, sampling port 1, f) Column 3, sampling port 1.

Interestingly, the measured solids concentrations in the settling column experiments did not vary significantly over time, and neither did the ratio of TS to VS (Fig. 6).

### 3.2 Tests with synthetic faeces in conical collection tank

#### 3.2.1 Visual observations

Similarly to the tests using real faeces, the synthetic faeces did not settle completely in any of the experiments using toilet paper (Figure d, supplementary material), which seemed to keep even those faeces without additional baker's yeast in suspension. Without toilet paper, only faeces containing baker's yeast floated. Left over night, these floating faeces would settle, i.e. reproducing a phenomenon in synthetic faeces previously observed in real faeces by Cruddas et al. (2015). For the lower volume-setting, the addition of toilet paper meant that most of the tank volume was filled by faeces and toilet paper, which absorbed a lot of liquid. Whether toilet paper was added or not, the lower volume tests resulted in a visibly higher solids to liquids ratio in the tank, and therefore better solid-liquid separation through displacement of liquid.

#### 3.2.2 Liquid sample measurements

TS, COD, and sCOD measurements generally rose over time (Figures f, g, and h in the supplementary material, respectively). With only four data points per experiment, any statement about the shape of the trend would be speculative. For the purposes of the statistical analysis, the trends were considered linear within the investigated range. Table 2 lists the mean slopes of the linear fit functions for the measured curves.

**Table 2:** Mean slopes ± standard deviation of the linear fit functions for the measured curves of TS, COD, and sCOD at the high (+) and low (−) settings for the three factors Tank Volume (V), Toilet Paper (TP), and Loading Frequency (F) in this order.

V/TP/F	TS	COD	sCOD
+ + +	94 ± 78.3	121.3 ± 75.1	73.9 ± 77.8
+ + −	25 ± 14.5	33.8 ± 19.3	13.1 ± 8.8
+ − +	172.7 ± 47.5	208.8 ± 95.5	53.8 ± 58.8
+ − −	125.6 ± 62.1	194 ± 94.7	28 ± 18.7
− + +	495.6 ± 209.7	497 ± 202.6	458.1 ± 202.6
− + −	860.1 ± 134.3	776.7 ± 169.2	719.5 ± 131.2
− − +	720.3 ± 139.4	995.5 ± 655.2	335.7 ± 131
− − −	425.5 ± 383.9	512.4 ± 341.5	270.1 ± 328.5

The Three-Way ANOVA yielded that the only statistically significant factor affecting the liquid quality paramters TS, COD, and sCOD was the liquid volume in the tank, V (*p* ≤ 0.05), while TP, F, or any interactions of the factors had no significant effect on either of the three parameters.

## 4 Discussion

Both in the settling columns with real faeces and in the conical tank with synthetic faeces, settling did not occur completely. This corresponds to our observations during field tests and confirms the need for additional processes to achieve solid-liquid separation. Toilet paper seems to be the crucial component preventing the settling of solids. And while toilet paper is not used everywhere in the world, with some people using other types of paper, and many using water, it can be expected that a significant portion of NMT users may, at some point, use toilet paper and input it into the NMT. Therefore, its effects on settling should not be ignored. With its low dry density of 260 g/l (Durukan and Karadagli, 2019), toilet paper should float. However, once soaked with water, it appears to remain at a static water level in the column and create a matrix sufficiently strong to keep faeces in suspension. Potentially, gases exiting the faeces (Levitt and Duane, 1972) were trapped under toilet paper, creating the visible “ballooning” effect. Theoretically, toilet paper is designed to disintegrate easily through water movement in the sewers, even though this does not always happen sufficiently well (Elmas and Ozturk, 2019; Eren and Karadagli, 2012). Field tests of a different onsite sanitation system encountered a similar problem with toilet paper: A conveyor belt system designed to separate solids from liquids as form of pre-treatment repeatedly jammed and even broke because of not disintegrated toilet paper (Sahondo et al., 2019). In our experiments, no stirring was carried out that could have caused toilet paper disintegration, as currently, the NMT's collection tank operates without stirring. However, ongoing research into maceration of faeces might prove that it is advantageous for auger-transport and dewatering, which could solve the problem of intact toilet paper. The experience of Sahondo et al. (2019), however, could also indicate that, when used, toilet paper may cause complications for maceration within the NMT.

The observations in both sets of experiments were distinctly different from settling processes in municipal wastewater or faecal sludge treatment: Toilet paper and faeces were still intact and did not settle well. The conical tank appeared to promote better settling than the vertical columns, which corresponds to the use of this shape in municipal treatment (Dodane and Bassan, 2014; Metcalf and Eddy Inc. et al., 2014). However, this observation will have to be validated using real faeces. While settling in septic tanks does not frequently appear to be a subject of research, the breakdown of toilet paper seems to be a consideration for their users nonetheless, as can be deduced from the existence of *septic tank safe toilet tissue* (Freedom Living, 2020). The present study would implicate that toilet paper that does not disintegrate is likely to not settle well, thus inhibiting the settling processes in septic tanks and other small-volume sanitation systems.

Displacement was shown to promote solid-liquid separation in the NMT: By lowering the liquid overflow level, a smaller maximum liquid volume was created, thus lowering the ratio of liquids to solids in the tank at a given time. Therefore, more liquid flows out of the tank into downstream membrane treatment processes, which is tantamount to achieving better phase separation. However, a too high solids content in the cylindrical settling columns caused the creation of a plug, under which further liquids were trapped. While the use of a conical tank in the NMT is likely to reduce the risk of plugs forming, there is still a chance for ‘bridging’ to occur, a type of clogging observed for example in grain silos (Wu et al., 2009). This could be exacerbated by the high stickiness of faeces, and their tendency to adhere to even smooth surfaces (Wang et al., 2019). Therefore, it may be worth investigating whether a higher liquid content would reduce the risk of bridging. As can be seen in water flush toilets, liquid is a good medium to greatly reduce the adhesion of faeces to smooth surfaces. Therefore, some liquid might have to remain in the tank. Future research could investigate bridging of real faeces in the NMT collection tank, and whether liquid content affects the occurrence of bridging.

The fact that TS and the ratio of TS to VS did not change significantly over time in the settling column experiments can likely be explained by the experimental procedure: In this set of experiments, both liquids and solids were continuously added to an originally empty container. When pouring liquids into the container, especially when faeces were at a higher level than liquids, small particles would be washed away and be suspended in the liquid fraction. Thus, the concentration of solids particles was quite high from the first measurement onwards. In the experiments with the conical tank, only solids were dropped into the tank initially filled with clear water. In this case, the originally very low solids content of the liquid samples rose over time (Figure f in supplementary material). While this disparity was not an expected outcome, it seems plausible that a similar process of *washing out* particles from faeces could occur in the NMT during normal use if faeces are not completely covered by liquid. This would be a reason to operate a small-volume collection tank like the NMT's with a high liquid to solids ratio, i.e. a higher overflow weir.

It seems unsurprising that measured COD concentrations rose steadily across all samples in both sets of experiments, save for some potential outliers. The longer faeces and urine remain mixed, the more organic compounds could dissolve into the liquid phase. This has implications for the operation of the collection tank in the NMT: To reduce the organic burden on the downstream membrane treatment processes (Kamranvand et al., 2018), it appears desirable to reduce contact time between faeces and urine, either by removing faeces from the tank as quickly as possible, or by lowering the liquid outflow point as much as possible, effectively draining the liquid quicker. Removing the faeces quickly would require more frequent operation of the auger, which requires energy. As the NMT is designed to be energy self-sufficient, higher energy consumption is undesirable. A lower outflow, and consequently lower liquid content in the collection tank would not require additional energy but might result in bridging. Hence, where on the spectrum between the two modes of operation – frequent emptying or low liquid overflow – the optimum will lie depends on the requirements for the auger to perform, whether a macerator is added to the tank, and whether bridging is likely to occur with real faeces. Current thinking on next steps in NMT design does indeed include a macerating blade on the auger to encourage solids pick-up by the screw and reduce the risks of blockages caused by paper and undigested solids (Ravndal et al., 2019). Ongoing NMT research will yield insights into these questions which have been informed by these settlement and displacement experiments.

The statistical analysis of the second dataset yielded the conclusion that, other than time, only the effective tank size had a significant effect on the quality of the overflowing liquid. This conclusion seems plausible: As suspected, the higher liquid volume appears to dilute the concentration of solid particles and organic load. Thus, the larger the liquid volume, the lower the pollution effect from a given amount of faeces would be. This would indicate that operating the tank with a high liquid volume and frequent solids removal might be favourable. This would not require more liquid to be treated by the downstream membrane processes, as only the overflowing liquid is treated. However, a higher weir-level in the tank would increase the time required to fill the tank with urine, faeces and toilet paper until the weir is reached. Thus, a longer contact time of faeces and urine is to be expected, which could result in a higher degree of pollution in the overflow liquid.

Surprisingly, the addition of toilet paper did not have a significant impact on the overflow liquid quality. A possible explanation for this could be that the suspension of faeces by toilet paper did not increase the faeces-urine interface and therefore the transfer of TS and COD into the liquid phase significantly.

The lack of a significant effect of loading frequency on overflow liquid quality may be explained by relatively short times for the physical processes in the conical tank to return to a static state. It may be that only high loading frequencies of several defecation events per hour significantly affect transfer of TS and COD into the liquid phase.

Considering that toilet paper did not affect overflow liquid quality, but the use of toilet paper does appear to cause suspension of solids that would otherwise settle, it seems advisable to avoid the use of toilet paper in the NMT. However, this would either add a burden to the solid waste collected in the toilet room or require a significant behaviour change for a large number of potential users, who habitually wipe rather than wash. As shown in the case of UDDT, behaviour change in toilet use is likely to meet opposition and may prevent the adoption of new sanitation technologies (Mkhize et al., 2017; Roma et al., 2013). Anal cleansing through washing would also add more liquid to the toilet, which would then need to be treated. Maceration of the tank's contents could facilitate settling but would likely result in higher TS and COD concentrations in the liquid phase. Advising the use of toilet paper that disintegrates easily, like *septic tank safe toilet tissue*, may be another approach to promote settling in the NMT's collection tank.

## 5 Conclusions

The presented research confirmed the casual observation of previous testing that settling of faeces and toilet paper in urine does not occur to a satisfactory degree in containers of similar volume and geometry to that of the NMT's collection tank. Toilet paper could be identified as instrumental inhibitor to settling and thus to effective solid-liquid separation.

A lower liquid overflow level could be shown to result in better solid-liquid separation in the tank. However, the study also concluded that contact time and liquid volume were affecting the quality of the overflowing liquid, and a higher liquid volume in the tank resulted in better quality overflowing liquid. These contradicting results could not be reconciled definitively.

In fact, it is practically impossible to decide how to best operate the NMT's collection tank without considering the NMT holistically. The NMT is a complex product consisting of multiple sub-products, or sub-systems, that affect and interact with one another. Before the subsystems can be integrated into a complete system to test all interactions in a fully functioning physical prototype, it is sensible to test the sub-systems individually and understand how they operate depending on various inputs. The present study has been such a sub-system test and only provides some information on which the decision of tank operation will be based. It is one of many prototype experiments required to develop a product as complex as the NMT.

## CRediT authorship contribution statement

**Jan Hennigs:** Conceptualization, Data curation, Formal analysis, Investigation, Methodology, Visualization, Writing - original draft, Writing - review & editing. **Kristin T. Ravndal:** Methodology, Writing - review & editing. **Alison Parker:** Conceptualization, Supervision, Writing - review & editing, Funding acquisition. **Matt Collins:** Conceptualization, Funding acquisition. **Ying Jiang:** Conceptualization, Funding acquisition. **Athanasios J. Kolios:** Conceptualization, Funding acquisition. **Ewan McAdam:** Conceptualization, Methodology, Funding acquisition. **Leon Williams:** Conceptualization, Funding acquisition. **Sean Tyrrel:** Conceptualization, Supervision, Project administration, Writing - review & editing, Funding acquisition.

## Declaration of competing interest

The authors declare that they have no known competing financial interests or personal relationships that could have appeared to influence the work reported in this paper.

## Appendix A Supplementary data

Supplementary material Image 1
