# Supplementary material for: Faeces – Urine separation via settling and displacement: Prototype tests for a novel non-sewered sanitation system
Source: Sci Total Environ. 2021 Jan 20;753:141881. doi: 10.1016/j.scitotenv.2020.141881 (PMC7674630; doi:10.1016/j.scitotenv.2020.141881)
Supplement: Supplementary file 1 — Supplementary material [file mmc1.docx]

**Supplementary Material**

Figure a: Average simulated toilet use events per day.


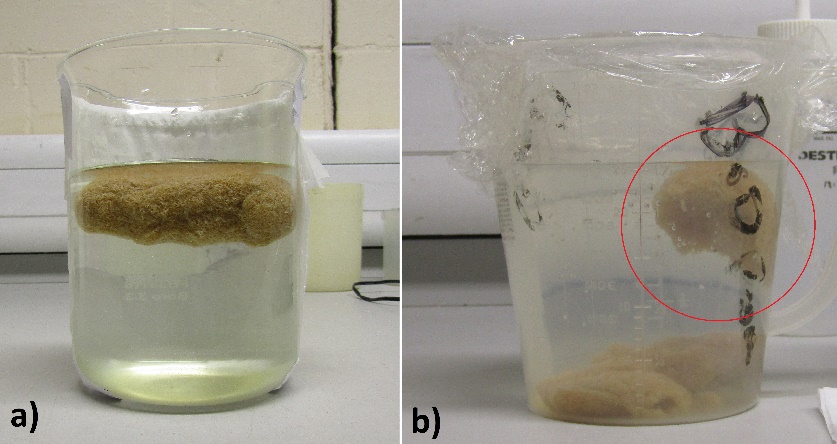


Figure b: Floating synthetic faeces. a) with 65% water content; b) with 80% water content - only partially floating.


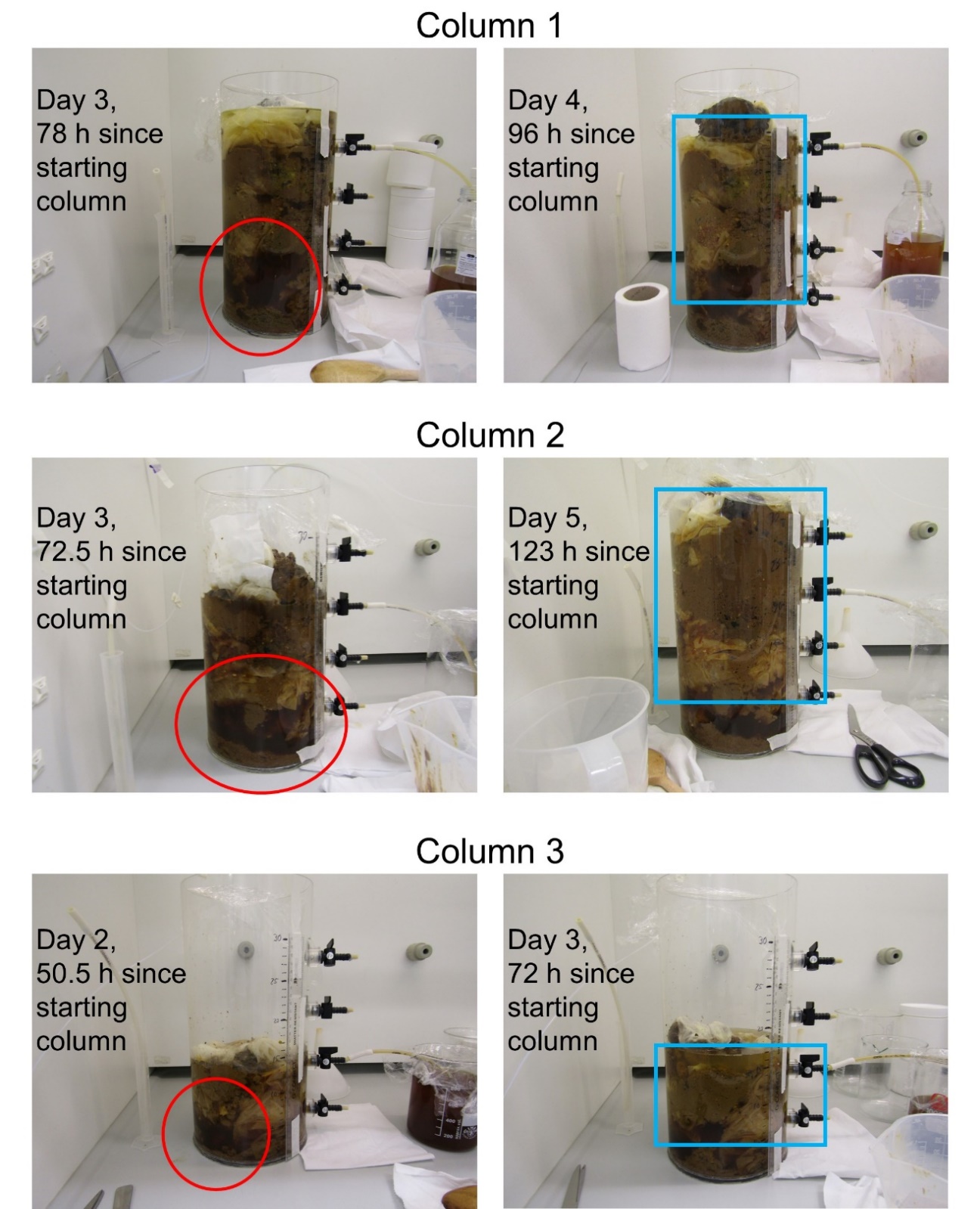


Figure c: Settling columns filled with real faeces and toilet paper on the last test day respectively. All three columns show distinctly that solids did not fully settle, but rather leave "pockets" of liquid (red circles) that are eventually covered by a dense layer of faeces (blue squares).


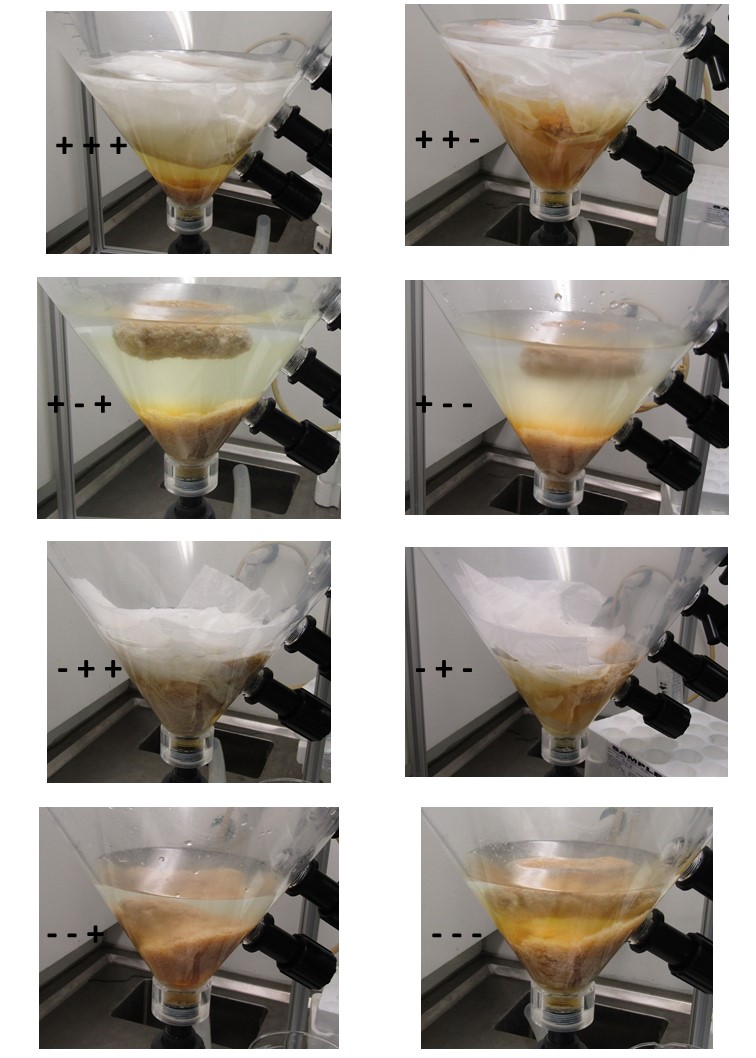


Figure d: images of the prototype tank at the end of an experiment for each level setting of the three factors V, TP, and F (+ and - symbols referring to the high and low levels of the three factors in this order).

Table a: Number of defecations and urinations during a given hour for the three column tests.

| **C1** | | | |  | **C2** | | | |  | **C3** | | | |
| --- | --- | --- | --- | --- | --- | --- | --- | --- | --- | --- | --- | --- | --- |
| **Day** | **Hour starting at** | **No. of faeces** | **No. of urine-loads** |  | **Day** | **Hour starting at** | **No. of faeces** | **No. of urine-loads** |  | **Day** | **Hour starting at** | **No. of faeces** | **No. of urine-loads** |
| 1 | 07:00 | 2 | 2 |  | 1 | 06:00 | 3 | 3 |  | 1 | 07:00 | 1 | 1 |
|  | 08:00 |  | 5 |  |  | 07:00 |  | 1 |  |  | 08:00 | 2 | 2 |
|  | 11:00 |  | 7 |  |  | 08:00 | 2 | 5 |  |  | 10:00 | 1 | 4 |
|  | 13:00 | 1 | 2 |  |  | 11:00 |  | 2 |  |  | 11:00 |  | 6 |
|  | 15:00 |  | 1 |  |  | 12:00 | 2 | 4 |  |  | 13:00 | 1 | 2 |
| 2 | 07:00 | 3 | 6 |  |  | 14:00 | 1 | 9 |  | 2 | 07:00 | 2 | 6 |
|  | 08:00 | 1 | 3 |  | 2 | 07:00 | 2 | 6 |  |  | 10:00 |  | 2 |
|  | 09:00 |  | 2 |  |  | 10:00 | 3 | 4 |  |  | 12:00 | 2 | 3 |
|  | 10:00 | 1 | 3 |  |  | 13:00 | 1 | 8 |  |  | 13:00 | 1 | 3 |
|  | 11:00 | 1 | 1 |  | 3 | 08:00 | 2 | 6 |  | 3 | 07:00 | 2 | 4 |
|  | 12:00 |  | 3 |  |  | 12:00 | 1 | 5 |  |  | 08:00 |  | 2 |
|  | 15:00 |  | 1 |  |  | 13:00 | 1 | 2 |  |  | 10:00 |  | 1 |
| 3 | 07:00 | 2 | 4 |  | 4 | 07:00 | 3 | 9 |  |  | 12:00 | 2 | 4 |
|  | 11:00 | 1 | 3 |  |  | 08:00 |  | 1 |  |  | 13:00 |  | 1 |
|  | 13:00 | 1 | 3 |  |  | 12:00 | 1 | 2 |  | 4 | 07:00 | 2 | 3 |
|  | 15:00 | 1 | 2 |  |  | 13:00 |  | 1 |  |  | 08:00 |  | 3 |
| 4 | 07:00 | 1 | 1 |  | 5 | 07:00 | 2 | 2 |  |  | 09:00 |  | 1 |
|  | 08:00 |  | 2 |  |  | 08:00 | 1 | 5 |  |  | 11:00 | 1 | 2 |
|  | 12:00 | 3 | 6 |  |  | 10:00 |  | 4 |  |  |  |  |  |
|  | 13:00 |  | 4 |  |  | 12:00 | 2 | 4 |  |  |  |  |  |
| 5 | 06:00 | 1 | 2 |  |  |  |  |  |  |  |  |  |  |
|  | 08:00 |  | 2 |  |  |  |  |  |  |  |  |  |  |
|  | 10:00 |  | 2 |  |  |  |  |  |  |  |  |  |  |
|  | 11:00 |  | 1 |  |  |  |  |  |  |  |  |  |  |
|  | 12:00 | 2 | 3 |  |  |  |  |  |  |  |  |  |  |

**Statistical Analysis**

**Levene’s Test of Equality of Error Variances** (Levene, 1960) based on the median showed that the assumption of equal variances was not violated for any of the three dependent variables (**Table b**), allowing for the ANOVA to be conducted.

**Table b: Results of Levene's Test of Equality of Error Variances based on the median.**

|  | Levene Statistic | df1 | df2 | Sig. |
| --- | --- | --- | --- | --- |
| TS | .644 | 7 | 17 | .714 |
| COD | .835 | 7 | 17 | .573 |
| sCOD | .741 | 7 | 17 | .641 |

Significant **ANOVA** **results** are shown in **Table c**. Only those effects of factors and interactions on independent variables are shown that displayed a high statistical significance (p < 0.05). These results indicate that TS are dependent on V, an interaction of TP and F, and a three-way interaction between V, TP, and F. COD appears to be dependent only on V, whereas sCOD appears dependent on V, TP, and the interaction of both. However, the two-way interaction of TP and F could, in turn, be moderated by V.

**Table c: Tests of Between-Subject Effects of the Three-Way ANOVA.**

|  | Dependent Variable | Type III Sum of Squares | df | Mean Square | F | p | Partial Eta Squared | Noncent. Parameter |
| --- | --- | --- | --- | --- | --- | --- | --- | --- |
| V | TS | 1679966.401 | 1 | 1679966.401 | 54.228 | 0.000 | 0.761 | 54.228 |
|  | COD | 1913974.459 | 1 | 1913974.459 | 24.642 | 0.000 | 0.592 | 24.642 |
|  | sCOD | 1009187.501 | 1 | 1009187.501 | 40.125 | 0.000 | 0.702 | 40.125 |
| TP | sCOD | 128834.797 | 1 | 128834.797 | 5.122 | 0.037 | 0.232 | 5.122 |
| V * TP | sCOD | 124304.731 | 1 | 124304.731 | 4.942 | 0.040 | 0.225 | 4.942 |
| TP * F | TS | 157852.444 | 1 | 157852.444 | 5.095 | 0.037 | 0.231 | 5.095 |
| V * TP * F | TS | 179141.424 | 1 | 179141.424 | 5.783 | 0.028 | 0.254 | 5.783 |
| Error | TS | 526654.063 | 17 | 30979.651 |  |  |  |  |
|  | COD | 1320392.946 | 17 | 77670.173 |  |  |  |  |
|  | sCOD | 427568.483 | 17 | 25151.087 |  |  |  |  |

Splitting the dataset into those values with V = + and those values with V = -, and a subsequent Two-Way ANOVA for the interaction of TP with F reveals that this interaction is moderated by the third factor V. While there is a significant effect for the interaction of TP * F at low V (p = 0.034), there is none at high V (p = 0.755). The plots for the estimated marginal means illustrate this clearly (**Figure c**). Therefore, the effect of this two-way interaction is not considered to be significant.


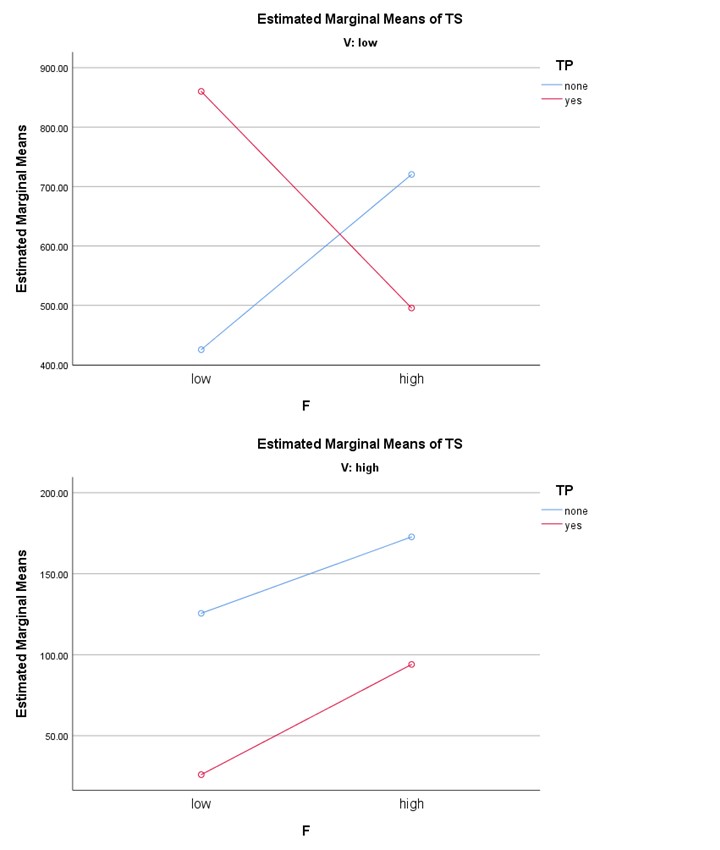


**Figure e: Plots of estimated marginal means of TS for the interaction of TP and F, and low and high levels for V, respectively. While the plot lines cross for low V, indicating an interaction, they do not cross for high V.**

When reviewing the estimated marginal means for the effects shown in **Table c**, it is revealed that the 95% confidence intervals for the high and low settings of the effects overlap in most cases (**Table d**), indicating that one cannot distinguish clearly between the levels of TP, the two-way interactions of V*TP and TP*F, and the three-way interaction of V*TP*F. The effect of these factors and interactions is therefore not considered to be significant. The intervals are distinct from each other only for the different levels of V, which is consequently left as the remaining significant factor affecting TS, COD, and sCOD in the overflowing liquid.

**Table d: 95% Confidence Intervals for the estimated marginal means of the dependent variables at the different levels of the significant effects. In most cases, the intervals overlap.**

|  |  |  | 95% Confidence Interval | |  |
| --- | --- | --- | --- | --- | --- |
| Effect | Dep. Variable | Level | Lower Bound | Upper Bound | Overlap |
| V | TS | - | 521.579 | 729.169 | No |
|  |  | + | -2.637 | 211.762 |  |
| V | COD | - | 531.020 | 859.718 | No |
|  |  | + | -30.272 | 309.205 |  |
| V | sCOD | - | 352.325 | 539.371 | No |
|  |  | + | -54.403 | 138.778 |  |
| TP | sCOD | - | 75.314 | 268.494 | Yes |
|  |  | + | 222.608 | 409.654 |  |
| V * TP | sCOD | - * - | 166.301 | 439.499 | Yes |
|  |  | - * + | 461.019 | 716.572 |  |
|  |  | + * - | -95.691 | 177.507 |  |
|  |  | + * + | -93.132 | 180.066 |  |
| TP * F | TS | - * - | 123.922 | 427.128 | Yes |
|  |  | - * + | 294.924 | 598.129 |  |
|  |  | + * - | 291.452 | 594.658 |  |
|  |  | + * + | 152.955 | 436.577 |  |
| V * TP * F | TS | - * - * - | 211.081 | 639.879 | Yes |
|  |  | - * - * + | 505.918 | 934.715 |  |
|  |  | - * + * - | 645.748 | 1074.545 |  |
|  |  | - * + * + | 309.878 | 681.227 |  |
|  |  | + * - * - | -88.828 | 339.969 |  |
|  |  | + * - * + | -41.662 | 387.135 |  |
|  |  | + * + * - | -188.435 | 240.362 |  |
|  |  | + * + * + | -120.419 | 308.378 |  |


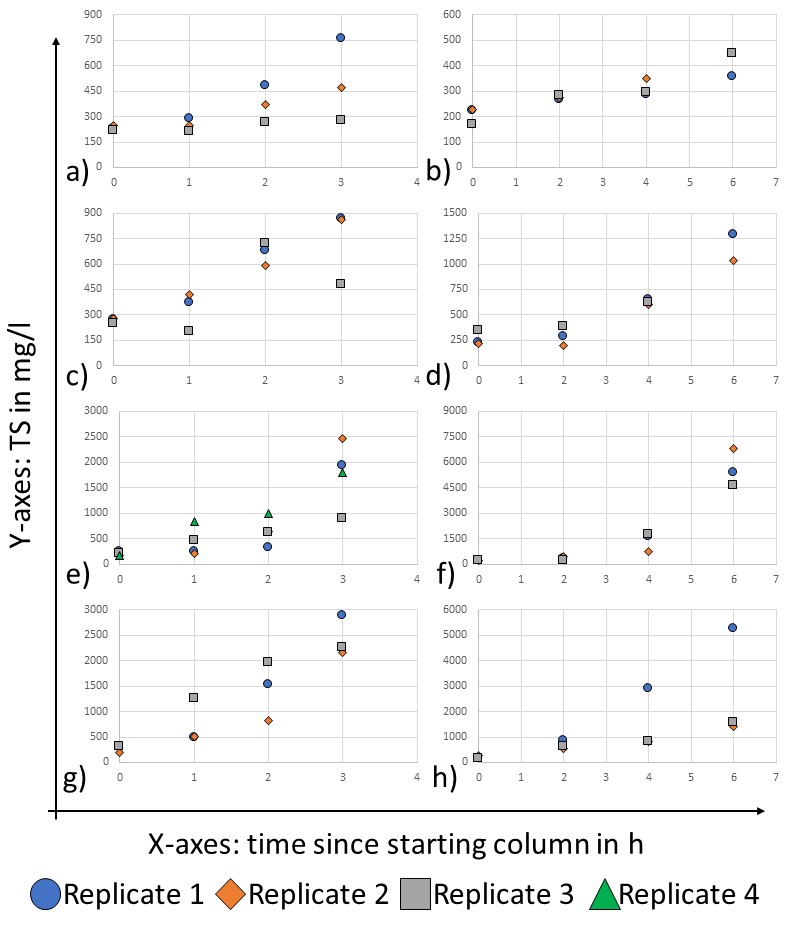


Figure f: TS measurements for conical tank tests. a) +++, b) ++-, c) +-+, d) +--, e) -++, f) -+-, g) --+, h) --- (+ and - symbols referring to the high and low levels of the three factors V, TP, and F in this order). Note the varying scales on the y-axes, made necessary to depict the trends.


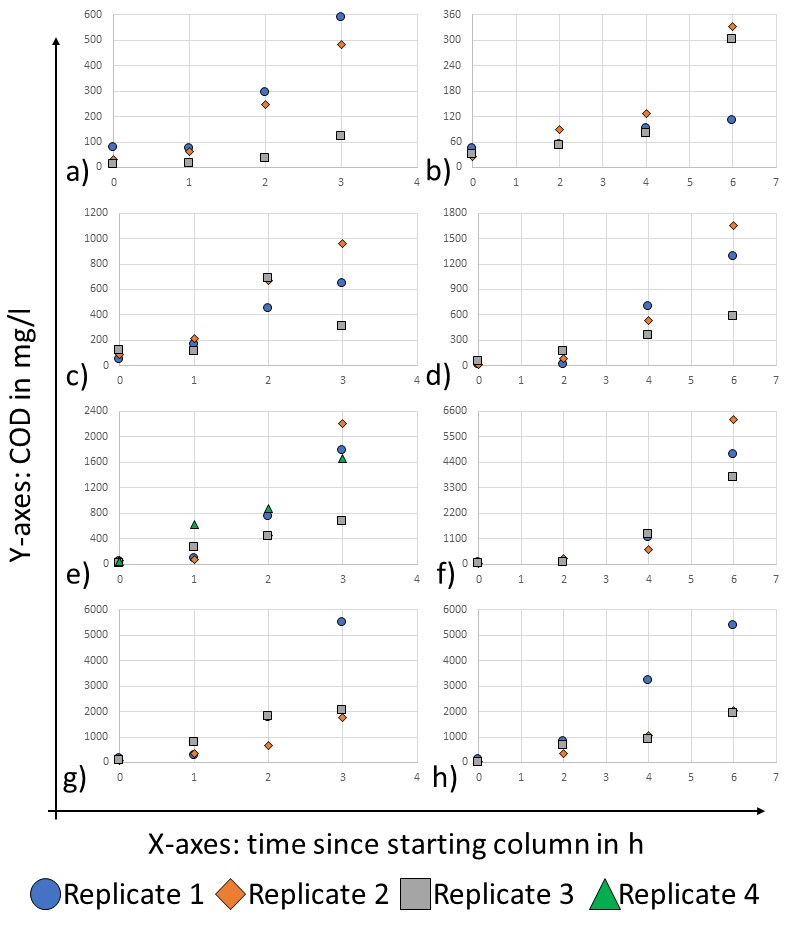


Figure g: COD measurements for conical tank tests. a) +++, b) ++-, c) +-+, d) +--, e) -++, f) -+-, g) --+, h) --- (+ and - symbols referring to the high and low levels of the three factors V, TP, and F in this order). Note the varying scales on the y-axes, made necessary to depict the trends.


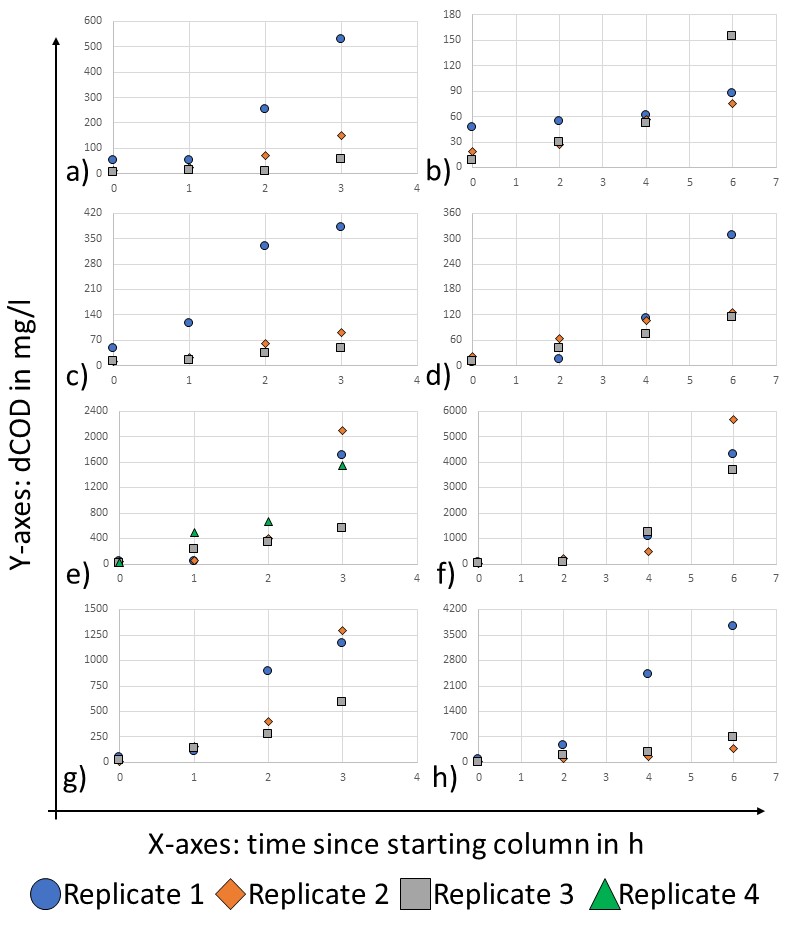


**Figure h: sCOD measurements for conical tank tests. a) +++, b) ++-, c) +-+, d) +--, e) -++, f) -+-, g) --+, h) --- (+ and - symbols referring to the high and low levels of the three factors V, TP, and F in this order). Note the varying scales on the y-axes, made necessary to depict the trends.**
